# Evaluation of dog owners' perceptions concerning radiation therapy

**DOI:** 10.1186/1751-0147-51-19

**Published:** 2009-04-29

**Authors:** Nanna Åkerlund Denneberg, Agneta Egenvall

**Affiliations:** 1 Jönköping Small Animal Hospital Oskarshallsgatan 6, SE-553 03 Jönköping, Sweden; 2 Department of Clinical Sciences, Faculty of Veterinary Medicine and Animal Husbandry, Swedish University of Agricultural Sciences, SE-750 07 Uppsala, Sweden

## Abstract

**Background:**

External radiation therapy (RT) has been available for small animals in Sweden since 2006. This study was designed to obtain information on owner experiences and perceptions related to RT of cancer in their dogs. Another survey was used to determine the attitudes about use of RT in a group of Swedish veterinarians. Their responses were analyzed and compared to their level of knowledge of oncology and RT.

**Methods:**

Owners of all dogs (n = 23) who had undergone RT for malignancy at Jönköping Small Animal Hospital between March 2006 to September 2007 were interviewed. A questionnaire was given to a selected group of veterinarians.

**Results:**

All 23 owners responded. All owners thought that their dog did well during RT and most that their dog was also fine during the following phase when acute RT-related skin reactions occur and heal. Three owners stated that their dog had pain that negatively impacted quality of life because of radiation dermatitis. Five owners reported that RT positively impacted quality of life of the dog during the first weeks after RT because palliation was achieved. The owners were not disturbed by the efforts required of them. All but one owner (22 of 23) stated that they would make the same decision about RT again if a similar situation occurred. The most important factor for this decision was the chance to delay occurrence of tumour-related discomfort. The chance for cure was of less importance but still essential, followed by expected side effects. Time commitments, travel, number of treatments required and financial cost; all had low impact. The veterinarian survey showed that less background knowledge of small animal oncology/RT was associated with more negative expectations of RT for small animals.

**Conclusion:**

The results show that for these owners, RT was a worthwhile treatment modality and that the discomfort for the dog was manageable and acceptable relative to the benefits. Improved continuing education about small animal RT in Sweden will likely result in increased evidence-based and positive treatment recommendations concerning RT by veterinarians.

## Background

Treatment with external beam radiation is one of the cornerstones in human cancer therapy. Among dogs, cancer was the most common specific cause of death or euthanasia as registered by a Swedish animal insurance company. Of all deaths with a diagnosis, 18% had cancer [1]. In veterinary medicine, radiation therapy (RT) has been used for the past 70 years and the science of RT is continuously developing [2-4]. The availability is still not worldwide but has increased in several industrialized countries. There were over 60 veterinary RT facilities in North America in December 2007 according to the Veterinary Cancer Society's homepage [5]. In Europe this number was only 8 (personal communication, Prof. Dr med vet B Kaser-Hotz, Diplomate ACVR, ECVDI Animal Oncolocy and Imaging Center Hünenberg, Schwitzerland). Thus RT, together with chemotherapy, are, in many countries, well accepted and commonly used treatment modalities for cancer in pets. In Scandinavia although surgery for cancer is well established, external RT has not been accessible and chemotherapy (except for the use of mitotane) has been regularly utilized in only a limited numbers of clinics and mainly during the last decade (Personal communication, Oncology Professor AT Kristensen, Faculty of Life sciences University of Copenhagen and Dr V Kristiansen, Norwegian school of Veterinary Sciences, Oslo). Systemic RT using radio-iodine has been available in Sweden for the treatment of hyperthyroid cats [6].

In February 2006, an orthovoltage machine was installed at one animal hospital in the south of Sweden (Jönköping Small Animal Hospital). This machine is dedicated to treat small animals with external beam RT. During the initial stages of offering a novel treatment modality, it is unlikely that veterinarians and owners will be fully aware of the limits and benefits of the new technique. With respect to cancer there are emotional factors that influence treatment decisions, in addition to the medical facts, among both owners and many veterinarians. It was therefore deemed worthwhile to document perceptions of owners and veterinarians on RT at the start of its introduction in Sweden.

Owner satisfaction with therapy has been studied for several disease complexes [7-10]. With respect to cancer, data on owner satisfaction with limb amputation has been reported [11]. Perceptions about chemotherapy have been studied in dog and cat owners [12]. In both studies it was shown that most owners were content with the chosen treatment for their animal.

The primary aim of this study was to obtain information on experiences and perceptions of owners that chose to have their dog undergo RT for cancer. The secondary aim was to describe attitudes about RT in a group of Swedish veterinarians, taking into account their level of knowledge of this modality and related issues.

## Materials and methods

### Animals

All dogs that received RT for malignancy (n = 23) from March 2006 to September 2007 were included

### Radiation therapy protocol

To achieve full immobilization during RT, dogs were sedated with a combination of medetomidine and butorphanol at the lowest effective dose. After RT, sedation was antagonized by atipamezol. In a few instances, for example, when the dog was experiencing temporary gastrointestinal disturbances, another form of anesthesia was used, i.e. a combination of acepromacine, buprenorphine and propofol.

The RT was administered using a Siemens Stabilipan ortovoltage teletherapy unit, operated at 20 mA and 190 kV, 145 kV and 110 kV, filtered with 0.5 mm Cu, 4 mm Al and 1 mm Al respectively. Focal spot to skin distance was 50 or 60 cm depending on required depth dose. Radiotherapy planning was by manual methods using single enface or parallel-opposed fields. Radiotherapy field sizes (side of square field) varied from 2 to 22 cm. Blocking in the field by lead rubber (4 mm lead rubber = 2 mm Pb) or lead eye lenses (2–3 mm Pb) were used in some cases. The stated doses are mid-tumour doses when either parallel opposed fields were used or when gross disease was treated with single lateral fields. Stated doses are surface doses or depth dose at 1 cm when single en face fields were used for microscopic disease.

In general, dogs suffering from a macroscopic tumour not amenable for surgery were given coarsely fractionated RT with a palliative intent. Dogs with microscopic residual disease after tumour surgery and a good prognosis were given standard (fine) fractionated therapy with curative intent. Acute radiation toxicity was assessed by the clinician using the VRTOG scoring scheme [13] (additional file 1).

### Medication

Prednisone, famotidine or chemotherapy was used in conjunction with RT in a few cases because of the biologic behavior of the specific tumour. All side effects were treated symptomatically when they occurred. Antibiotics, non steroidal anti inflammatory drugs, prednisone and occasionally butorphanol sublingual tablets were prescribed when needed. Some of the dogs that had grade 3 cutaneous reactions [13] were managed with hydrogel (Intrasite, Smith-Nephew) and a moisture vapor permeable dressing (Allevyn, Smith-Nephew) during their maximal phase of acute toxicity.

### Questionnaire design

A questionnaire was developed to explore owner's perceptions and experiences from having their dog treated with RT for malignancy (tables 1a, b, and 2, translation from the Swedish original). The questionnaire was pre-tested by a few veterinarians and after minor changes also by 3 owners who had their dog treated with chemotherapy. Based on their comments the questionnaire was slightly modified. The final version included a total of 31 closed questions (tables 1a and 1b) and, at 3 locations requests for further comments. In another 8 questions, owners were asked to grade their answers (writing a number from 0 to 10) with endpoints defined as in table 2. It was the subjective perception of the owner that was being targeted throughout the questionnaire.

**Table 1:** **a Responses to the owner questionnaire, categorical answers**.

Question	Category	Number	%
**1) Opinions on the dog and family members related to radiation treatment (RT).**			
How did I learn about RT as an available treatment option for my dog	Advice of my primary vet	20	87
	Second opinion of another veterinarian	2	9
	Trough media/internet	1	4
I had previous knowledge about RT in humans	Yes	9	39
	No	14	61
How did I decide to use RT for my dog?	My own decision	6	26
	Consensus of family members in favor	17	74
	Family disagreed	0	0
My dog had veterinary care insurance that covered most of the cost for RT	Yes	9	39
	No	14	61
**2a) Describe your impression of the well-being of your dog during the weeks while RT was performed**			
My dog appeared well during the time of treatment	Yes, very well	15	65
	Yes, fairly well	8	35
	Did not appear well	0	0
My dog found it unpleasant to visit the hospital	Yes	3	13
	Yes a little	11	48
	No	9	39
My dog appeared tired during the treatment	Yes	5	22
	Yes a little	6	26
	Not tired	12	52
Did I notice a change in the dog's quality of life?	No Impact	19	83
	Positive impact	3	13
	Negative impact	1	4
**2b) Describe what happened during the 4 weeks following completion of RT. (This is when acute skin and/or mucosal effects occur and heal).**			
My dog appeared well during these weeks	Yes, very well	14	61
	Yes, well	6	26
	No, not well	3	13
My dog experienced discomfort in the radiation field	Yes severe	5	22
	Yes, minor	10	44
	No discomfort seen	8	35
My dog was in pain during these Weeks	Yes, severe	3	13
	Yes, modest	6	26
	No	14	61
My dog experienced side effects	Yes, severe	5	22
	Yes, minor	10	44
	No	8	35
During post treatment, did the quality of life of the dog seem different?	No impact	15	65
	Positive impact	5	22
	Negative impact	3	13

**b Responses to owner questionnaire, categorical answers**.			

Question	Category	Number	%

**3) Describe your own perception or personal experience from when your dog had RT:**			
I believe that RT cured my dog's cancer	Yes	9	39
	No	11	48
	Can't decide	2	9
I believe that RT does prolong life until tumour related discomfort occur	Yes	21	91
	No	2	9
My dog's clinical signs disappeared because of RT: (limited to 14 dogs with gross disease)	Yes, completely (n = 14)	5	36
	Yes, in part (n = 14)	4	29
	No (n = 14)	5	36
	NA^a ^(subclinical disease) (n = 23)	9	39
I believe I was adequately informed about the RT before starting RT	Yes	22	96
	No	1	4
The information was consistent with the outcome for the dog	Yes	21	91
	No	2	9
The information agreed with what I experienced	Yes	22	96
	No	1	4
My dog experienced more severe side effects than I expected	Yes	5	22
	No	18	78
My dog experienced fewer side effects than I expected	Yes	14	61
	No	9	39
I was well informed how to care for side effects	Yes	19	83
	No	0	0
	NA	4	17
Management of side effects was effective	Yes	10	43
	Yes in part	5	22
	No	0	0
	NA	8	35
**4) Describe the level of satisfaction with RT in your dog.**			
RT was worth my commitment in time, travel, caring for the dog at home etc.	Yes	23	100
	No	0	0
Subjecting my dog to RT was worth my financial cost	Yes	22	96
	No	1	4
I consider that the discomfort to my dog due to RT was worth the gain	Yes	23	100
	No	0	0
I would use RT again in a similar situation with another dog.	Yes	22	96
	No	0	0
	Missing	1	4

^a ^NA- not applicable

**Table 2:** Responses to the owner questionnaire, ordinal and continuous answers.

						Number of owners with a response										
Question	Mean	STD	Median	Min	Max	0	1	2	3	4	5	6	7	8	9	10
**1^b^. How many people were there in the household?**	2.6	1.2	2	1	6	-	-	-	-	-	-	-	-	-	-	-
How many dogs were there in the household?	1.7	0.7	2	1	3											
How many cats were there in the household?	0.7	1.2	0	0	4											
**2^b^. My overall impression how RT was experienced by my dog (quality of life), from initiating RT until side effects had declined.**						0 = good quality- 10 = severe negative impact on dog										
						
	2.9	2.4	2	0	8	4	3	6	4	0	2	1	2	1	0	0
								
**3^b^. Reactions from acquaintances when informed that my dog had undergone RT for cancer.**				Percent		0	10	20	30	40	50	60	70	80	90	100
				
Positive reactions %	76.9	29.5	90	0	100	1	0	2	0	0	2	0	4	2	4	8
Negative reactions %	14.3	19.8	10	0	80	10	5	4	2	0	1	0	0	1	0	0
Neutral or No reaction %	11.8	26.8	0	0	100	18	2	0	0	0	1	1	0	0	0	1
						0 = Calm, confident – 10 = Very stressful and inconvenient										
						
Overall: My own experience from start of RT until side effects declined	2.3	1.9	2	0	6	5	3	7	2	1	4	1	0	0	0	0
**4^b^. Would I choose RT again in a similar situation?**																
Grade the importance of following factors deciding to treat with RT or not						0 = no importance, negligible- 10 = crucial influence, very important.										
						
My financial cost^a^	2.2	2.6	1	0	8	9	3	2	1	2	3	0	0	2	0	0
Prognosis: chance for cure^a^	7.3	3.1	8	0	10	2	0	0	0	1	4	0	2	3	2	8
Prognosis: chance for longer life without tumour related discomfort^a^	9.5	1.1	10	6	10	0	0	0	0		0	1	1	1	2	17
Severity of expected side effects^a^	6.6	2.4	7	1	10	0	1	0	2	0	5	2	4	3	2	3
Number of treatments required^a^	3.0	2.8	2	0	10	6	1	5	2	1	4	1	1	0	0	1
My investment in time and travel	2.3	2.7	2	0	10	9	1	4	2	0	3	1	1	0	0	1

^a ^one response missing, ^b ^for titles of sections 1–4, see tables 1a and 1b

The questionnaire consisted of four sections (tables 1a, b and 2). The purpose of the first section was to cover the background, obtaining information about how the owner was informed that RT was a possible option, the composition of the household of the dog at the time and how the decision to have the dog undergo RT was made. The second (dog) section investigated the experiences of the dog and its quality of life during RT and for the first month thereafter. The third (owner) section explored how the owners felt about having their dog treated with RT, whether therapy was beneficial and reactions from other people who learned the dog had received RT. The final section evaluated owner satisfaction with the outcome, if it was worth the effort required and if they would make the same decision again if a similar situation occurred with another dog. Owners were also asked to evaluate the importance of various factors on that decision (table 2)

The printed questionnaire was mailed together with an explanatory covering letter to all owners during October 2007. It was completed during a telephone interview (author NÅD) during November 2007.

A short questionnaire was constructed to explore the attitudes towards use of RT among a convenience sample of Swedish veterinarians. The following questions were asked to define the veterinarian's knowledge of RT: time since graduation, present occupation and whether he/she had attended lectures on small animal clinical oncology and/or small animal RT. The respondents were asked to grade their opinions on 4 different issues using a number from 0 to 10. The issues were if small animal RT is justified, what is the well-being of the dog during RT, if RT is a hazard for personnel and how often RT is indicated in a clinical setting. In three of the questions, 10 was defined as the most positive rating but in the question concerning potential risk for personnel, 10 was defined as the most severe risk. Voluntary additional comments were encouraged. Veterinarians attending three professional meetings (a course on cytology for practitioners, a congress of Swedish dermatologists and a seminar on the ethics of canine oncology) in Sweden were invited to participate, anonymously. Eighty-two questionnaires were distributed.

### Data analysis

Results of the owner survey are presented using medians, ranges, means and standard deviations, for continuous and score variables. The qualitative variables are presented using numbers and percentages.

Results of the veterinarian questionnaire are presented as median, ranges and means. Respondents were categorized into two groups based on their stated knowledge of RT; one category that responded that they had not attended lectures in small animal oncology or clinical RT contrasted to those that had attended lectures in at least one of the subjects. The scores from the four questions were compared using the Wilcoxon signed test. The two-sided p-value limit was set < 0.05.

## Results

### Animals

Of the 23 dogs, 4 had died or been euthanized when the interview was conducted. At the time of treatment the median age was 8 years (range 2–13). Gender distribution was 8 intact females, 13 intact males, 1 spayed female and 1 castrated male. Breeds included 3 golden retrievers, 2 Labrador retrievers, 2 miniature schnauzers and one of each the following breeds: flatcoated retriever, Bernese mountain dog, Swedish elkhound, rottweiler, whippet, bearded collie, wirehaired dachshound, boxer, Airdale terrier, German shepherd, Shetland sheepdog, Danish-Swedish farm dog.

Twelve dogs had undergone incomplete tumour resection prior to referral and 3 of these suffered from gross progressive disease. Information on histological diagnoses, total doses of radiation and type of fractionation are summarized in table 3.

**Table 3:** Diagnosis and type of radiotherapy (RT) in the 23 dogs in the study: Radiation toxicity score and number of dogs dead at interview are also shown.

Diagnosis	Site	Standard fractionation RT^a^ No. of dogs (score^b^)	Coarse fractionation RT^c^ No. of dogs (score^b^)
Mast cell tumour all grades	Extremities (n = 2) Head (n = 3)	2 (1,1)	3 (1*, 1, 2)
Oral malignant melanoma	Cheek (n = 1) Pharynx (n = 1)		1 (1*) 1 (0)
Mucosis fungoides	Head		1 (0)
Hemangiopericytoma	Extremities	2 (1,3)	2 (2, 3)
Fibrosarcoma	Maxilla	1 (1)	
Undifferentiated sarcoma	Axilla		1 (0*)
Chondrosarcoma	Maxilla		1 (3)
Osteosarcoma	Appendicular		1 (3)
Cutaneous angiomatosis	Metatarsus		2 (1, 3)
Sweat gland carcinoma	Extremities		2 (2, 3*)
Adenocarcinoma	Nasal cavity		1 (0)
Squamous cell carcinoma	Nasal cavity		1 (2)
Thyroid carcinoma	Ventral neck		1 (0)

^a ^RT in 3–4 Gy fractions, total dose 48–54 Gy.

^b ^VROTG radiation morbidity score (see additional file 1, LaDue T, Klein MK. Toxicity criteria of the veterinary radiation therapy oncology group. Veterinary Radiology & Ultrasound 2001;42:475–476).

^c ^RT in 5–10 Gy fractions, total dose 24–36 Gy.

* Dead or euthanized at the time of interview.

### Radiation therapy

A single field was used in 18 cases and parallel-opposed fields in 5 cases. Four dogs received 16–18 fractions of 3–3.5 Gy delivered 4 days a week over a period of 25 days for postoperative microscopic disease, resulting in total doses from 48 to 54 Gy. One dog received, after cytoreductive surgery, 12 fractions of 4 Gy 3 days a week over a period of 25 days, for residual macroscopic tumour.

For 18 dogs, coarse fraction schemes were used, administrating 3 fractions of 8–10 Gy on a day 0-7-21 schedule (5 dogs), 4 weekly fractions of 8 Gy (4 dogs) or 5–6 fractions of 5–6 Gy 1–2 times a week (9 dogs) resulting in total doses from 24 to 36 Gy. Of these 18 dogs, 12 dogs received RT with a palliative intent for advanced local disease not amenable to surgery and in one dog as pain management for appendicular osteosarcoma.

One dog suffered from localized mucosis fungoides and the remaining four dogs were treated with coarsely fractionated therapy for postoperative microscopic disease instead of regular fractionation because of other considerations (high probability of metastatic dissemination, the owner considered the dog too old or because coarse fractionation techniques have been reported superior for the actual diagnosis (Oral malignant melanoma: [14-16])). All but 3 dogs were brought to the hospital for RT on treatment days. Of the 3 dogs that were hospitalized, two dogs stayed at home during weekends.

### Medication

One dog with pharyngeal oral malignant melanoma had the primary tumour removed prior to RT and also received carboplatin (300 mg/m^2 ^and 270 mg/m^2 ^respectively, as intravenous infusions during 5–10 minutes) in conjunction with the second and sixth fraction of RT. This dog developed myelo-suppression as a result of chemotherapy and this fact impacted its quality of life. The two dogs that had macroscopic mast cell tumours were medicated with prednisone and famotidine 1–2 weeks prior to initiation of RT, during, and 1 month after RT.

### Radiation Toxicity

Grading of acute radiation morbidity, according to the VROTG scheme [13] (additional file 1), assessed by the clinician, revealed moderate toxicity with mean and median scores of 1.6 and 1. These average scores included 6 dogs with grade 3 toxicity in which confluent moist dermatitis was present (table 3).

If separated into regular fractionation treatment and coarse fraction treatment, the average toxicity score was 1.4 for regular fraction compared to 1.5 in the coarse fraction group.

Two of the dogs with grade 3 toxicity had received coarse fraction treatment for a tumour for which the symptoms were hard to distinguish from those of radiation toxicity since the original lesion was moist and irritated (sweat gland adenocacinoma, cutaneous angiomatosis). Another dog in the coarse fraction group with grade 3 toxicity had extensive self-inflicted trauma by licking prior to assessment of toxicity.

### Dog owners' questionnaire

All 23 owners responded, all questionnaires but one were complete and the results are shown in tables 1a, b and 2. The time span from completion of RT until the interview varied from 1 to 19 months (data not shown). All owners thought that their dog appeared well during RT, even though they noticed some tiredness or nervousness. During the first 4 weeks after completion of RT, when acute radiation dermatitis typically culminates and then heals, 13% of owners considered that their dog had pain that negatively impacted quality of life. A positive influence of RT on quality of life was reported by 22% since the palliative treatment had diminished existing inconvenience from the tumour. The overall impression of how RT was experienced by the dog, was rated 2 (median) on a scale (0–10) where 0 was defined as the best quality of life (table 2). Owners were satisfied with the information given before and during RT and felt that the outcome was well-correlated to their expectations. For example, the median rating of the owners' overall experience from initiating RT until side effects declined was 2 (0 equals calm and confident and 10 very stressful (table 2)).

Owners were not disturbed by efforts required of them and all owners, except one, would make the same decision about RT again if a similar situation occurred (tables 1b and 2). The most essential factor for this decision was the chance to prolong the duration of freedom from disturbing tumour-related clinical signs. The chance for cure was of less weight but still important, followed by the extent of expected side effects. The commitment of time and effort and cost had low impact among these owners (table 2). The voluntary additional comments were sparse but positive. It was often stated that this care had been better and more compassionate compared to human healthcare.

### Questionnaire to veterinarians

Sixty-seven of the 82 questionnaires (81.7%) distributed to veterinarians were returned. Of the responding veterinarians, 20 had not attended education programs on veterinary oncology or RT, whereas 47 had studied one or both of the subjects. The scores of positive attitude towards RT were lower (p = 0.01) in the less-informed group with a median score of 5 (range 0–10, mean 4.5) compared to the median 7.5 (range 1–10, mean 6.9) among those who had sought more information on cancer therapy. Assumptions about the quality of life of dogs treated with RT (where 0 equals bad and 10 equals good) was also different (p = 0.003) between the two groups with a median score of 6 (range 2–10, mean 6.5) in the better-informed group, compared to 5 (range 0–8, mean 4.6) among those who had not attended the mentioned lectures. With regard to whether RT is a hazard to personnel, there was no significant difference between the groups (p = 0.37) with a median of 5 for both. (Mean score for assumptions of danger (0 equals no hazard, 10 equals severe danger) was 4.5 (range 0–10) in the less-educated group compared to 3.6 (range 0–8) in the other.) Clinical indications for RT were considered to occur slightly more often (p = 0.03) by the informed veterinarians with a median of 5 (range 0–9, mean 5.4) compared to the less-informed group, median 5 (range 0–7, mean 4.2) (0 equals never indicated).

## Discussion

It was encouraging to a veterinarian pioneering the use of RT in Sweden, that these owners, that choose to have RT performed on their dogs, believed RT had been a worthwhile treatment modality and that the discomfort for the dog seemed manageable and acceptable in relation to the health benefits. All owners found RT worth the efforts required of them and all but one would make the same decision again. (The only exception was an owner that did not respond because she was too old to have another dog in her lifetime). However, these respondents were not a random sample of Swedish animal owners with dogs suitable for RT, but a biased group that had decided to use RT on their dogs. They took this decision in spite of cost, travel and other inconveniences. Nevertheless, if the experience had been very negative, they presumably would have expressed that on the questionnaire. A survey of reluctant owners that actually declined RT may have given different results and reasons why they choose not to use RT.

Most owners perceived their dog's quality of life to be unaffected or improved during the weeks of RT. One dog that deteriorated also had carboplatin infusions and suffered from myelo-suppression which undoubtedly contributed to the reduced quality of life. The majority of owners also thought that the acute side effects were well-tolerated by their dog. Every owner stated that the inconvenience for the dog was acceptable in relation to the gain. The time span from completing RT until the interview was performed was long for some owners and this may have affected the owner response, provided the impression of various experiences may change with time. Also, if owners had an unstated bad conscience for putting their dog through painful side effects, they may have repressed their true perceptions and recalled a false positive experience. However, in general, the clinicians' assessments of morbidity correlated with owner impressions (data not shown). The average score of acute side effects [13] in the studied group was modest, likely reflecting the high percentage of cases receiving a palliative regimen. The confusing fact that average and median scores of radiation toxicity were higher in the coarse fraction group (1.5 and1.5) compared to the regular fraction group (1.4 and 1) may be a function of difficulties in assessing toxicity when the original lesion was moist and irritated. If these 2 cases and also the self-inflicted trauma case were excluded from the group of animals that received coarse fraction treatment, average and median scores (1.2 and 1) would be closer to the expected outcome. Small sample size also gives these uncommon cases high impact on the outcome.

Few dogs were subjected to RT because of microscopic disease (4 dogs that received standard fractionation, 3 dogs that received coarse fractionation). The indications for curative intent RT may have been less well known during these initial 19 months with access to RT in Sweden. Thus most referred cases were advanced and not amenable for surgery.

There are limitations with this study. The sample size was small and the patient material was heterogeneous with different diagnoses, locations and other considerations leading to different total doses, used kV, field sizes, fraction sizes, intervals between fractions and medications among the animals. This prohibits the data from being used as an evaluation of treatment efficacy or whether the dogs had more side effects than was acceptable in correlation with outcome (a difficult task with a short follow-up). However, the aim of this study was to seek the owner perspective in the available group of clients.

The telephone interviews were conducted by the clinician in charge (NÅD) of the performed RT. There may be a loyalty-based relationship between this clinician and the owner although owners were repeatedly encouraged to be totally honest in their responses. This design may have produced bias in form of "too positive" responses. On the other hand, this intimate setting may have facilitated retrieval of specific information and also may have contributed to the 100% response rate among owners. Using telephone interviews was to be one way of preventing misunderstandings on both sides.

A rough attempt was made to make a judgment of the dogs' quality of life through a few owner questions. In contrast, there are in human cancer care questionnaires such as the "European Organization for Research and Treatment of Cancer 30-item core quality of life questionnaire" (EORTC QLQ-30), a validated, standardized and copyrighted instrument that is used in more than 3000 studies worldwide [17,18]. It is supplemented by disease specific modules e.g. breast, lung, head and neck cancer. These are used to assess the influence of side effects on quality of life among groups of patients with specific diagnoses. However, assessments are subjective also from standardized and validated questionnaires, since the information relies on self-reported data [19]. The EORTC QLQ-30 should be used to retrieve information only on subjective side effects and more quantitative evaluation should be used for objective changes. One cannot conclude from one to the other since patient assessments of symptoms have been shown to be poor predictors of objective findings [19,20]. In veterinary medicine, it has been shown that few dog-owners were able to distinguish the various grades of lameness in cases of cranial cruciate ligament rupture and repair. An inconsistency was found between owner perception of lameness and results of force plate analysis performed on dogs that underwent surgery for this problem. However, owner assessment was concluded useful for estimating functional outcome of cruciate repair, but should not replace professional judgment [21,22].

The overall value of owner opinion surveys can be questioned. The issue whether another person (proxy) other than the patient, is able to judge quality of life for the patient has been investigated in several human studies [23-25]. When patient, spouse and treating physician completed the same quality of life questionnaire, there was substantial variability between ratings of quality of life by physicians or partners, as compared to patient ratings [23]. In a similar triple survey concerning patients that had undergone anterior skull base surgery, the operating surgeon ratings had no correlation with patient ratings, with surgeons overestimating patients' quality of life while there was a significant overall agreement between patient and partner responses [24]. In children too young or too severely ill to answer a questionnaire, parent proxy-reports may be the only available information. Several reports of inconsistent proxy-report between parent and pediatric patient have been presented [25-27] but statistical strategies to examine proxy agreement in quality of life have not reached consistency [25].

Owners' statements about their pet are also subjective proxy-reports. There may be misjudgments on pain or discomfort. However, when overall quality of life during a defined time span is evaluated, the owner is the one who spends most of their time with the pet and knows the habits of their dog. Owners' proxy assessments complement the professional evaluation made by veterinary surgeons, when a true estimation of quality of life is the goal.

The questionnaire to veterinarians was limited to volunteers, clearly being a convenience sample. The attendees at the 3 meetings were not representative of Swedish small animal veterinarians in general. However, interestingly significant differences were found in 3 of 4 questions posed. Veterinarians that had attended education on clinical oncology or small animal RT had more positive attitudes than those less well-informed. This could indicate a pre-existing skepticism until evidence-based information is gained. The result could also be interpreted that these negative veterinarians had made active decisions not to become informed because of a pre-existing standpoint that RT for animals is unethical, as some of them commented in the questionnaire. The veterinarians assumptions of quality of life of dogs submitted to RT were investigated: with 0 being the best quality of life, owners score was rated 2 (median) compared to score of 4 and 5 (median) among informed and less-informed veterinarians respectively. Thus according to the owners, well-being of the dog submitted to RT was clearly more positive than these veterinarians thought it would be.

Among both categories of surveyed veterinarians, the perception of RT as a hazard was common. Personnel are never allowed to be in the treatment room during RT, and the walls of this room have to be in accordance with the legislation. Thus, when correctly performed, RT actually subjects health care workers to less radiation than ordinary X-ray imaging techniques, where people may get exposed to radiation, while holding the animals. Voluntary comments from responding veterinarians also indicated confusions between RT and chemotherapy and between external beam RT and systemic radio-nucleotide therapy or brachy-therapy. Thus some individuals seemed to believe that the patient becomes radioactive from external beam RT, or is shedding cytotoxic waste.

Availability of RT brings new possibilities of curing an unknown number of dog and cat patients and to relieve clinical signs from cancer in other animals, given that RT is considered early in the workup of oncology cases. For this to happen, awareness among veterinary practitioners about the main indications and limitations of RT is mandatory. Evaluation of owner experience from performed RT may, especially when the procedure is newly introduced (e.g. in a country), together with the numerous available scientific reports of efficacy and morbidity, assist owners and veterinary surgeons in the decision-making process in case of malignancy.

The goal of small animal veterinary decisions (not just oncological) should be that the dog can benefit from a good quality of life. To assess outcome, veterinarians will have to continue to rely on owner proxy-reports, together with more objective clinical assessments. Therefore the development of standardized and validated instruments for rating the quality of life of the animal is a field that deserves continuing interest.

## Conclusion

The owners of the first group of dogs treated with RT in Sweden, considered RT as a worthwhile treatment modality and that the discomfort for the dog seemed manageable and appropriate relative to the benefits. The findings from the small survey of veterinarians suggest that increasing the information about small animal RT and cancer therapy will improve veterinary attitudes about use of RT and other therapies.

## Competing interests

The author NÅD is employed by Jönköping Small Animal Hospital and is the clinician in charge of the clinical work performed at this RT facility. NÅD is also shareholder in Djursjukhuset i Jönköping AB. However, the present study and writing of manuscript have been performed during a leave of absence.

## Authors' contributions

NÅD was responsible for the design of the study and questionnaires, for the distribution and the retrieval of questionnaires and also performed the interviews. NÅD was responsible for interpretation of the data and writing the draft of the manuscript. AE contributed in the design of the study, performed the statistical analysis and revised the manuscript. Both authors have read and approved the manuscript.

## Supplementary Material

Additional File 1 **VROTG Acute radiation Morbidity Scoring Scheme**. Here the scoring scheme for radiation toxicity of skin and mucous membranes is shown. Click here for file
