# Supplementary material for: Evaluation of dog owners' perceptions concerning radiation therapy
Source: Acta Vet Scand. 2009 Apr 29;51(1):19. doi: 10.1186/1751-0147-51-19 (PMC2687441; doi:10.1186/1751-0147-51-19)
Supplement: Additional File 1 — VROTG Acute radiation Morbidity Scoring Scheme. Here the scoring scheme for radiation toxicity of skin and mucous membranes is shown. [file 1751-0147-51-19-S1.doc]

**Additional file**

**VROTG Acute Radiation Morbidity Scoring Scheme**

| Organ/tissue | 0 | 1 | 2 | 3 |
| --- | --- | --- | --- | --- |
| Skin/hair | No change over baseline | Erythema, dry desquamation, alopecia/epilation | Patchy moist desquamation without edema | Confluent moist desquamation with edema and/or ulceration, necrosis, hemorrhage |
| Mucous membranes/oral cavity | No change over baseline | Injection without mucositis | Patchy mucositis with patient seemingly pain free | Confluent fibrinous mucositis necessiting analgesia, ulceration, hemorrhage, necrosis |

Displayed are parts of the scores approved by the Veterinary Radiation Therapy Oncology Group with the intent to serve as standard guidelines in the field of veterinary oncology. The complete classification scheme for acute and late toxicity criteria can be found in: LaDue T, Klein MK. **Toxicity criteria of the veterinary radiation therapy oncology group.** Veterinary Radiology & Ultrasound 2001;**42**:475-476.
